# Does Patient-Reported Olfactory Improvement Correlate with Quality of Life and Polyp Size Reduction in Patients with CRSwNP Treated with Dupilumab?

**DOI:** 10.3390/medicines13030026

**Published:** 2026-08-24

**Authors:** Brian Hernández-Martín, Miriam del Carmen Marrero-Ramos, María Soledad Cabrera-Ramírez, Jesús Javier Benítez-Rosario, Carmen Delia Dávila-Quintana, María Sandra Domínguez-Sosa

**Affiliations:** 1 Departamento de Otorrinolaringología, Hospital Universitario de Gran Canaria Dr. Negrín, 35010 Las Palmas de Gran Canaria, Spain; 2 Departamento de Métodos Cuantitativos en Economía y Gestión, Universidad de Las Palmas de Gran Canaria, 35017 Las Palmas de Gran Canaria, Spain

**Keywords:** CRSwNP, type 2 inflammation, dupilumab, olfaction disorders

## Abstract

Background: Olfactory dysfunction (OD) is a key symptom of chronic rhinosinusitis with nasal polyps (CRSwNP). Dupilumab improves clinical outcomes, including patient-reported olfactory function. This study aimed to analyze correlations between patient-reported olfactory improvement, quality of life, and endoscopic outcomes in patients treated with dupilumab. Methods: A real-life, single-center retrospective study included 35 patients with severe, uncontrolled CRSwNP treated with dupilumab. Patient-reported olfactory function was assessed using a visual analog scale (VAS-olfaction), quality of life with the Sinonasal Outcome Test-22 (SNOT-22), and polyp size with the Nasal Polyp Score (NPS) at baseline, 6 months, and 12 months. Changes from baseline were analyzed using the Wilcoxon signed-rank test, whereas correlations were evaluated using Spearman’s rank correlation coefficient. Results: Dupilumab was associated with significant improvements in patient-reported olfactory function, quality of life, and polyp size at 6 and 12 months. Improvements in VAS-olfaction correlated significantly with SNOT-22 at 6 months (*p* = 0.035) and 12 months (*p* = 0.024). In a sensitivity analysis excluding the smell/taste item from the SNOT-22, these correlations were attenuated and no longer reached statistical significance. No significant correlation was observed between patient-reported olfactory improvement and NPS. Conclusions: Patient-reported olfactory improvement correlated with quality of life but not with reductions in global polyp burden. These findings suggest that this improvement may not be fully explained by changes in polyp size alone.

## 1. Introduction

Chronic rhinosinusitis with nasal polyps (CRSwNP) is a chronic inflammatory disease of the nasal mucosa and paranasal sinuses. It is identified clinically by the presence of persistent symptoms, such as nasal congestion, anterior or posterior rhinorrhea, facial pain/pressure, and olfactory dysfunction (OD). Endoscopic evidence of nasal polyps and/or the presence of sinus opacification on computed tomography (CT) is required for diagnosis [1].

CRSwNP has a real impact on health-related quality of life (HRQoL), affecting patients’ physical, psychological, and social domains. Furthermore, this impact may be worse in cases of comorbidities such as asthma and NSAID-exacerbated respiratory disease (N-ERD), or even in patients with a clinical history of numerous sinonasal surgeries [2].

The sense of smell plays an important role in human life. It helps us detect dangerous situations, such as smoke or toxic gases, but it also triggers memories and emotions. Hence, OD is one of the most disabling symptoms in CRSwNP. In addition to affecting quality of life, it is usually resistant to conventional treatment, and it is associated with severe disease. It can also be one of the first signs of recurrence [3].

The pathophysiology behind OD remains unknown, but two main mechanisms have been proposed. The first one corresponds to an obstructive problem, where nasal polyps hinder the arrival of odor molecules to the olfactory epithelium. The second one is related to a sensory problem, in which type 2 inflammatory cytokines, such as interleukin (IL)-4 and IL-13, exert a direct cytotoxic effect on the olfactory neuroepithelium [4].

In recent years, a better understanding of type 2 inflammatory mechanisms has led to a significant change in the management of CRSwNP. Biological therapies have opened new possibilities, and among them, Dupilumab stands out as one of the most effective treatments. By blocking IL-4Rα, a shared receptor for IL-4 and IL-13, this monoclonal antibody significantly reduces the main inflammatory cytokines involved in type 2 inflammation [5].

Several clinical trials and real-life studies have demonstrated the efficacy and safety of Dupilumab in CRSwNP patients [6,7,8,9]. It has been associated with a reduction in polyp size, improvement in sinonasal symptoms and, consequently, improvement in HRQoL [10].

Similarly, Dupilumab has shown significant early olfactory recovery, which is one of the first indicators of treatment effectiveness [11]. In addition, available studies have demonstrated sustained duration of olfactory recovery over time [12].

Despite this knowledge, few studies have examined the relationship between olfactory recovery, quality of life and reduction in polyp size in patients with CRSwNP [13]. Therefore, the objective of this study was to assess whether subjective olfactory recovery correlated with improvements in quality life and reductions in polyp size among patients treated with Dupilumab.

## 2. Materials and Methods

### 2.1. Study Design and Population

This was a retrospective, monocentric, real-life observational study that included severe and uncontrolled CRSwNP patients. All subjects were followed at the Otorhinolaryngology Department of a tertiary referral center in Spain.

Electronic medical records from the Airway Diseases Unit were reviewed between January 2022 and January 2023. The patients provided written informed consent before data collection. The study was approved by the Hospital Ethics Committee (CEIm code: 2023-053-1).

CRSwNP was diagnosed according to European Position Paper of Rhinosinusitis and Nasal Polyps (EPOS2020). Dupilumab 300 mg was administered subcutaneously once every 2 weeks, following the same guideline criteria [1]. Asthma was diagnosed using the 2022 Global Initiative for Asthma Strategy [14].

The study included 35 adult patients (>18 years old) with severe CRSwNP who completed 12 months of Dupilumab treatment. The inclusion criteria were as follows:

- Endoscopic nasal polyp score (NPS) ≥ 4 (minimum score of 2 in each nasal cavity);
- Sinonasal Outcome test (SNOT-22) ≥ 50;
- ≥1 endoscopic sinonasal surgery (ESS) in the last 10 years;
- Inadequate symptom control with standard of care and/or failure of previous ESS;
- Exceptionally, patients with medical contraindications to ESS and concomitant asthma.

Exclusion criteria included asthma exacerbation requiring hospitalization within 4 weeks prior to inclusion, immunosuppressive therapy before inclusion, or chronic corticosteroid therapy for chronic autoimmune disorders.

### 2.2. Study Methods

Demographic and clinical characteristics were recorded for all patients. These included age, sex, and smoking status, as well as relevant comorbidities such as asthma, atopy, N-ERD and previous ESS. The diagnosis of atopy was established by positive skin prick testing or elevated serum specific IgE levels, as described by the European Academy of Allergy and Clinical Immunology [15].

Oral corticosteroid (OCS) dependence was defined as the need for continuous or repeated courses of systemic corticosteroids during the year prior to Dupilumab initiation. The use of OCS during follow-up was recorded as rescue treatment at 6 and 12 months.

Clinical evaluations were conducted at baseline, before starting biological therapy, and after the first administration at 6 and 12 months. In each evaluation, three main parameters were prospectively assessed. Subjective olfactory function was evaluated using a visual analog scale (VAS-olfaction), ranging from 0 (no alteration) to 10 (complete loss of smell). HRQoL was measured by the Sinonasal Outcome Test–22 (SNOT-22), a validated questionnaire with scores ranging from 0 (no symptoms) to 110 (worst symptoms) [16]. Finally, polyps’ size was quantified endoscopically using the Nasal Polyp Score (NPS), with scores from 0 (no polyps) to 8 (complete obstruction) [17].

### 2.3. Statistical Analysis

Data were analyzed using IBM SPSS Statistics, v.26.0. Categorical variables are presented as frequencies (n) and percentages (%), while continuous variables are expressed as mean and standard deviation (SD) when the data adhere to a normal distribution.

Changes over time were analyzed using the Wilcoxon signed-rank test for paired samples. Comparisons were performed between baseline and 6 months, and between baseline and 12 months. For descriptive purposes, delta values (Δ) were calculated as follow-up score (6 and 12 months) minus baseline score for VAS-olfaction, SNOT-22, and NPS. Therefore, negative values indicate clinical improvement.

The normality of the data distribution was assessed using the Shapiro–Wilk test. As the main variables did not follow a normal distribution, non-parametric tests were used. Correlations between continuous variables were evaluated using Spearman’s rank correlation coefficient (ρ). Comparisons between independent groups (sex and smoking status) were performed using the Mann–Whitney U test.

To assess whether the observed association between changes in olfactory function and quality of life was influenced by the smell/taste item included in the SNOT-22 questionnaire, a sensitivity analysis was performed. For this analysis, the smell/taste item was excluded from the SNOT-22 total score, and correlations between changes in VAS-olfaction and the SNOT-22 total score excluding the smell/taste item were reassessed using Spearman’s rank correlation coefficient.

A *p* value < 0.05 was considered statistically significant.

## 3. Results

### 3.1. Demographic and Clinical Characteristics

We included 35 patients with severe CRSwNP who completed 12 months follow-up of Dupilumab treatment. No patients withdrew from the study. Patients’ characteristics at baseline are reported in Table 1.

Twenty-two patients (62.9%) were male. The mean age was 54.4 ± 11.2 years old. At baseline, all patients (100%) had asthma as a comorbidity, and fourteen (40%) met criteria for N-ERD. Sixteen patients (45.7%) had a positive history of perennial aeroallergen sensitization.

Thirty of our patients (85.7%) had previously undergone EES, of which twelve (34.3%) only once and eighteen (51.4%) two or more times. At baseline, 34 patients (97.1%) were dependent on OCS. During follow-up, the need for OCS markedly decreased. Two patients required OCS rescue treatment during the first 6 months of follow-up, and one patient required OCS rescue treatment between months 6 and 12, all because of asthma exacerbations. These courses were completed several months before the corresponding outcome assessments.

All patients continued treatment with intranasal corticosteroids and nasal saline irrigations throughout the study period. Dupilumab was generally well tolerated, and no severe adverse events were observed during follow-up.

None of the patients showed blood eosinophil counts ≥ 1500 cells/µL at 12 months of treatment, and no clinically relevant eosinophil-related complications were recorded.

### 3.2. Timeline of Clinical and Endoscopic Parameters

Follow-up data showed a noteworthy treatment effect across all study parameters. This improvement was evident at 6 months and was maintained at 12 months of follow-up. The evolution of each parameter is summarized in Table 2.

A significant improvement in olfactory function was observed following dupilumab treatment. At baseline, the mean VAS-olfaction score was 8.5. It decreased to 2.7 after 6 months of treatment (Δ 6 months-Baseline = −5.8, *p* < 0.001) and remained stable at 12 months, with a mean value of 3.0 (Δ 12 months-Baseline = −5.5, *p* < 0.001).

A marked improvement in HRQoL was also recorded. The mean SNOT-22 score decreased from 65 at baseline to 8.8 at 6 months (Δ 6 months-Baseline = −56.2, *p* < 0.001). This improvement was maintained at 12 months, with a mean score of 9.1 (Δ 12 months-Baseline = −55.9, *p* < 0.001).

Endoscopic outcomes showed a consistent reduction in polyp size. The mean NPS decreased from 5.6 at baseline to 1.2 at 6 months (Δ 6 months-Baseline = −4.4, *p* < 0.001) and remained stable at 12 months, with a mean value of 1.1 (Δ 12 months-Baseline = −4.5, *p* < 0.001).

The percentage of patients with severe nasal polyposis (NPS ≥ 4) was 85.7% (30/35). After 12 months of dupilumab treatment, this proportion decreased to 8.5% (3/35). Notably, despite the persistence of moderate-to-severe nasal polyposis in a subset of patients, a marked improvement in olfactory function was still observed.

### 3.3. Correlation Analysis of Main Parameters

**Figure 1:**
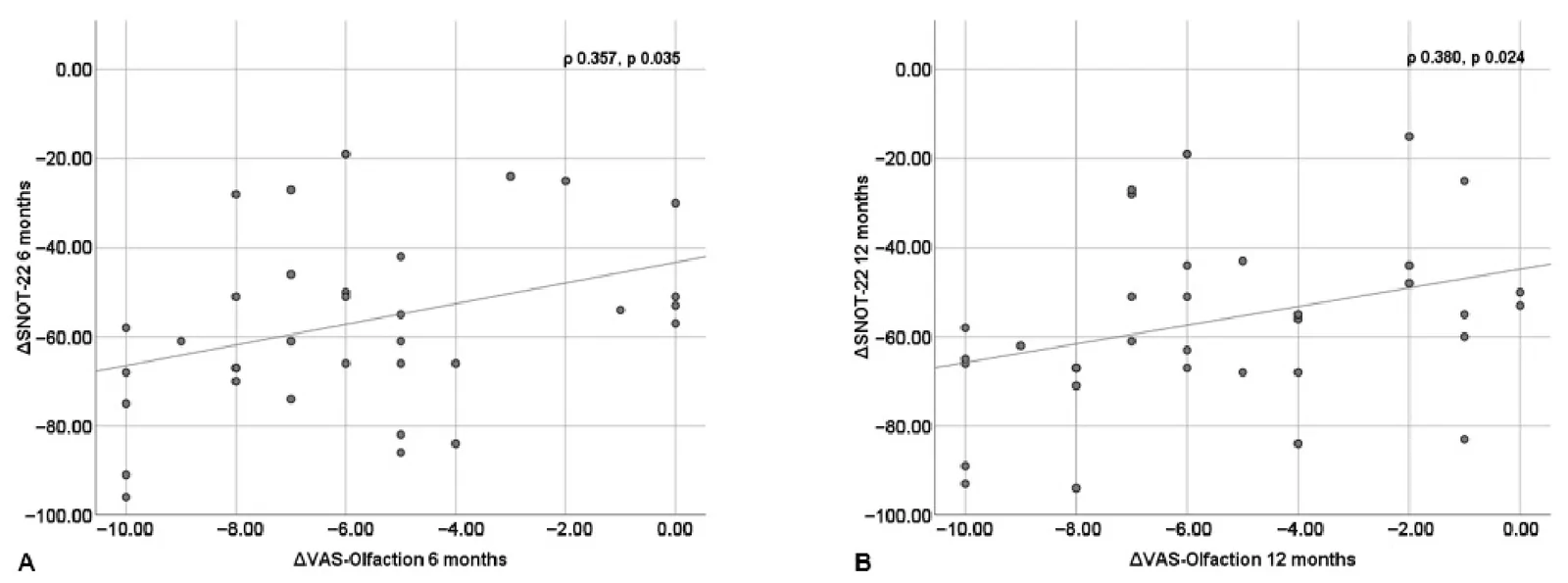
Scatter plots showing the correlations between changes in olfactory function (ΔVAS-olfaction) and changes in quality of life (ΔSNOT-22) at (**A**) 6 months and (**B**) 12 months. Delta values were calculated as follow-up minus baseline; therefore, negative values indicate clinical improvement. Lines represent the fitted Spearman regression trend.

To evaluate whether improvement in olfactory function was associated with changes in quality of life or polyp size, a Spearman correlation analysis was performed. The correlation coefficients and corresponding *p*-values are summarized in Table 3. Significant correlations between changes in olfactory function and quality of life at 6 and 12 months are illustrated in Figure 1.

Correlation analysis revealed a significant association between olfactory function and health-related quality of life. Changes in VAS-olfaction score correlated significantly with changes in SNOT-22 score both at 6 months (*p* = 0.035) and at 12 months (*p* = 0.024). In contrast, no significant correlation was observed between changes in VAS-olfaction score and NPS at either 6 months (*p* = 0.089) or 12 months (*p* = 0.084), indicating that improvement in olfactory function may occur independently of changes in polyp size.

To further evaluate whether the observed association between olfactory improvement and quality of life was influenced by the smell/taste item included in the SNOT-22, a sensitivity analysis was performed. After excluding this item from the SNOT-22 total score, the correlation between changes in VAS-olfaction and changes in the modified score was attenuated and no longer reached statistical significance at either 6 months (*p* = 0.090) or 12 months (*p* = 0.053).

No significant associations were found between changes in olfactory function and age, sex, or smoking status.

## 4. Discussion

OD is one of the most disabling symptoms of the CRSwNP, with a clear impact on safety, emotional well-being, and ultimately, HRQoL (3). Although it is recognized as a key symptom of CRSwNP, its pathophysiopathologic mechanisms and clinical implications have historically received limited attention. The COVID-19 pandemic marked a turning point, renewing scientific interest in olfactory function and stimulating research into the structural and inflammatory pathways that can compromise the olfactory neuroepithelium [18].

Despite its clinical relevance, olfactory recovery in CRSwNP remains one of the most challenging outcomes to achieve with conventional treatments. In this context, biologic therapies, particularly Dupilumab, have emerged as an effective alternative for this refractory symptom, showing more consistent and sustained improvements than those obtained with traditional therapies [10].

In our study, we observed a significant improvement in patient-reported olfactory function, accompanied by improvements in SNOT-22 and NPS at 6 months, which were maintained at 12 months of Dupilumab treatment. This pattern is consistent with previous reports and is further supported by the 24-month real-life data published by De Corso et al. In that study, the clinical, endoscopic, and olfactory response induced by Dupilumab were sustained over time and continued to improve in a proportion of patients. Notably, even “very late responders” showed additional benefits during the second year of treatment, highlighting the long-term durability of Dupilumab and supporting its maintenance beyond the first year in responding patients [19].

After analyzing the temporal evolution of the main variables, we evaluated whether improvements in patient-reported olfactory function correlated with improvements in HRQoL and with reductions in polyp size. We found that improvements in olfactory function were significantly correlated with improvements in HRQoL at both 6 months (*p* = 0.035) and 12 months (*p* = 0.024), suggesting a parallel evolution of these two outcomes. In contrast, no statistically significant correlation was observed between changes in VAS-olfaction scores and NPS at either 6 months (*p* = 0.089) or 12 months (*p* = 0.084). These findings suggest that improvements in olfactory function and reductions in polyp size may not occur in parallel, suggesting that olfactory recovery may be influenced by mechanisms beyond simple reduction in polyp burden.

When the smell/taste item was excluded from the SNOT-22 total score, the correlation coefficients were attenuated and no longer reached statistical significance. This finding suggests that part of the observed association may reflect overlap between the subjective olfactory assessment and the smell/taste domain of the SNOT-22 questionnaire. Nevertheless, the modest reduction in the correlation coefficients also suggests that the loss of statistical significance may be partly related to the limited sample size and the consequent reduction in statistical power.

To date, only a limited number of studies have jointly explored the correlations between olfactory function, HRQoL, and polyp burden. Among them, Cantone et al. [13] conducted a similar analysis with assessment at 3 and 6 months of Dupilumab. They reported a significant correlation between patient-reported olfactory improvement and HRQoL, while no significant correlation with NPS was observed. These results are consistent with our findings, which extend these observations to a 12-month follow-up period.

Similarly, Stilo et al. [3] specifically examined the relationship between olfactory function and polyp size, without finding significant correlation between these variables throughout follow-up. Together with our findings, these observations suggest that patient-reported olfactory improvement may not be fully explained by changes in global polyp burden alone, and that additional inflammatory and neurosensory mechanisms may also contribute to this process.

Current evidence supports that OD in CRSwNP arises from two main mechanisms. On the one hand, a conductive component, whereby sinonasal polyps and mucosal edema limit the access of odorant molecules to the olfactory epithelium. On the other hand, a neurosensory component related to type 2 inflammation affecting the olfactory neuroepithelium [20].

Chronic inflammation of the olfactory epithelium can impair neuronal function through apoptosis of olfactory sensory neurons and disruption of signal transmission. Several type 2 inflammatory cytokines, including IL-5 or IL-13, have been shown to be elevated in the olfactory cleft of patients with CRSwNP and to correlate inversely with olfactory function [20]. Moreover, tissue eosinophilia has been independently associated with olfactory loss, even regardless of polyp burden, reinforcing the role of inflammatory and neurosensory mechanisms in OD [21].

The mechanism of action of Dupilumab provides a plausible explanation for these findings. By blocking IL-4 and IL-13 signaling, key drivers of type 2 inflammation, Dupilumab reduces eosinophilic inflammation and modulates the inflammatory milieu of the nasal mucosa. In addition, experimental and translational data suggest that IL-4Rα receptor blockade may promote a more favorable environment for the regeneration of olfactory sensory neurons from basal cells, potentially contributing to olfactory recovery [4].

In our study, no significant correlation was observed between changes in patient-reported olfactory function and nasal polyp size. This finding suggests that the improvement in smell reported by patients treated with Dupilumab may not be fully explained by changes in polyp burden alone. Although our data do not allow us to determine the underlying mechanism, they are consistent with the hypothesis that modulation of type 2 inflammation and neurosensory pathways may play a relevant role in olfactory recovery.

Several limitations should be considered when interpreting these findings. This was a single-center study including a limited number of patients with severe and treatment-refractory CRSwNP, all of whom were treated with dupilumab, without a control group. Furthermore, our real-world cohort included patients with heterogeneous clinical characteristics within severe CRSwNP, which may limit the generalizability of these findings to the broader CRSwNP population. In addition, olfactory function was assessed using a subjective visual analog scale, without complementary psychophysical testing. These findings should be interpreted in light of these considerations.

## 5. Conclusions

Our data support the effectiveness of dupilumab in improving patient-reported olfactory function in patients with severe, uncontrolled CRSwNP in a real-life setting. Patient-reported olfactory improvement was significantly correlated with improvement in HRQoL, whereas no correlation was observed with reductions in nasal polyp size. These findings suggest that patient-reported olfactory recovery may not be fully explained by changes in global polyp burden alone. Accordingly, the beneficial effect of dupilumab on olfactory recovery may be related not only to polyp size reduction but also to its anti-inflammatory action and potential neurosensory restoration. In line with this, dupilumab may represent a well-tolerated therapeutic option for patients with uncontrolled CRSwNP.

## Figures and Tables

**Table 1:** Patients’ characteristics at baseline (*N* = 35).

Personal Characteristics	Mean ± SD	*N* (%)
Age (years)	54.4 ± 11.2	
Gender		
Male		22 (62.9)
Female		13 (37.1)
Current smokers		2 (5.7)
**Comorbidities and allergies**		
Asthma		35 (100)
N-ERD		14 (40)
Atopy		16 (45.7)
**Control of disease**		
Previous ESS		30 (85.7)
1 surgery		12 (34.3)
≥2 surgeries		18 (51.4)
OCS dependence		34 (97.1)
**Scores**		
VAS-olfaction score	8.5 ± 2.4	
SNOT-22 score	65 ± 21	
NPS	5.6 ± 1.7	

Data are expressed as absolute and relative percentage frequency for qualitative data, media, and SD for quantitative data. N-ERD: NSAID-exacerbated respiratory disease. ESS: endoscopic sinonasal surgery. OCS: oral corticosteroid. VAS: visual analog scale. SNOT-22: Sinonasal Outcome Test-22. NPS: Nasal Polyp Score.

**Table 2:** Evolution of clinical and endoscopic parameters at 6 and 12 months (*N* = 35).

Variable	Baseline	6 Months	ΔBL-6 Months	*p* Value	12 Months	ΔBL-12 Months	*p* Value
VAS-Olfaction score	8.5 ± 2.4	2.7 ± 2.8	−5.8	<0.001	3.0 ± 2.9	−5.5	<0.001
SNOT-22 score	65 ± 21	8.8 ± 7.1	−56.2	<0.001	9.1 ± 7.5	−55.9	<0.001
NPS	5.6 ± 1.7	1.2 ± 1.3	−4.4	<0.001	1.1 ± 1.3	−4.5	<0.001

Values are expressed as mean ± SD. Δ represents the change calculated as follow-up minus baseline; negative values indicate improvement. BL: baseline; VAS: visual analog scale; SNOT-22: Sinonasal Outcome Test-22; NPS: Nasal Polyp Score.

**Table 3:** Spearman’s correlation coefficients.

Correlation	Time Point	Spearman ρ	*p*-Value
VAS-olfaction score vs. SNOT-22 score	6 months	0.357	0.035
	12 months	0.380	0.024
VAS-olfaction score vs. SNOT-22 score (excluding smell/taste item)	6 months	0.291	0.090
	12 months	0.329	0.053
VAS-olfaction score vs. NPS	6 months	0.291	0.089
	12 months	0.297	0.084

Spearman’s rank correlation coefficient (ρ) and corresponding *p* values are reported. VAS: visual analog scale; SNOT-22: Sinonasal Outcome Test-22; NPS: Nasal Polyp Score.

## Data Availability

The original contributions presented in this study are included in the article. Further inquiries can be directed to the corresponding authors.

## Abbreviations

The following abbreviations are used in this manuscript:

OD	Olfactory dysfunction
CRSwNP	Chronic rhinosinusitis with nasal polyps
VAS	Visual analog scale
SNOT-22	Sinonasal Outcome Test-22
NPS	Nasal Polyp Score
CT	Computed tomography
HRQoL	Health-related quality of life
N-ERD	NSAID-exacerbated respiratory disease
IL	Interleukin
ESS	Endoscopic sinonasal surgery
OCS	Oral corticosteroids
SD	Standard deviation
